# Pyrrolizidine alkaloid contamination of food in Africa: A review of current trends and implications

**DOI:** 10.1016/j.heliyon.2024.e24055

**Published:** 2024-01-03

**Authors:** Emmanuel Letsyo, Felix Kwashie Madilo, Liticia Effah-Manu

**Affiliations:** Department of Food Science and Technology, Faculty of Applied Sciences and Technology, Ho Technical University, P.O Box HP 217, Ho, Ghana

**Keywords:** Africa, Pyrrolizidine alkaloid, Food contamination, Food safety, Toxicity, Honey

## Abstract

Pyrrolizidine alkaloids (PAs) contamination of foodstuffs has become a topical issue in recent years on account of its potential hepatotoxicity to consumers. This review therefore highlights human exposure to PAs across Africa, focusing on their occurrence, current trends of food contamination, and their associated health implications. A comprehensive search of peer-scientific literature and relevant databases, PubMed, Google Scholar, Science Direct, Web of Science and Scopus, was conducted from 2001 to 2023 focusing mainly on foodstuffs, including grains, herbs, teas, honey, and livestock products. The findings revealed that PA contamination is a prevalent issue in several African countries, with the primary sources of contamination attributed to the consumption of honey and the use of PA plants as herbs in food preparations. Additionally, poor farming practices have been found to influence the presence and levels of PAs in foodstuffs. To mitigate PA contamination in food and safeguarding public health across the continent, several strategies are proposed, including the implementation of stringent regulatory and quality control measures, adoption of Good Agricultural Practices, and public awareness campaigns to educate producers, consumers and beekeepers about the risks associated with PA-contaminated food products.

## 1 Introduction

**Fig. 1:**
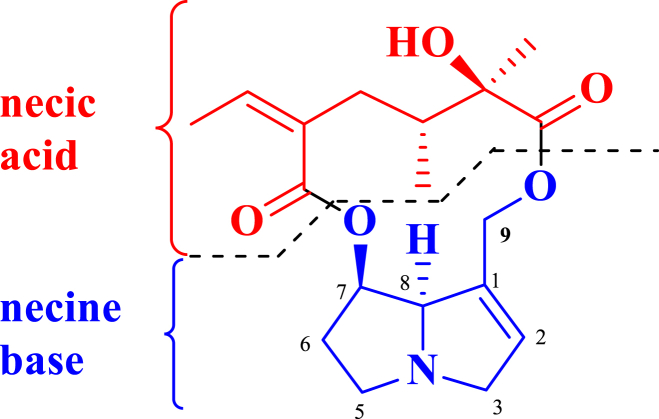
General pyrrolizidine alkaloid structure showing necine base and necic acid as well as the double bond at the 1,2-position of the necine base, which render it potentially hepatotoxic and/or carcinogenic.

In recent years, the issue of food safety has gained significant attention due to its profound impact on public health and well-being [1]. Among the various emerging food contaminants, pyrrolizidine alkaloids (PAs) and their oxidized forms (PA *N*-oxides, PANOs), specifically the 1,2 unsaturated ones, have emerged as a significant concern [2,3] on account of their liver-damaging and cancer-causing effects. PAs are protoxins produced primarily by 3 % of the world's flowering plants that act as a natural defense system for the producer plants against herbivores [2,4], while at the same time may become hepatoxic in both animals and humans when ingested [5,6]. The producer plants are predominantly found within Asteraceae (*Senecio*, *Eupatorium*, and other genera of the tribes Senecioneae and Eupatoriae), Boraginaceae (*Heliotropium*, *Trichodesma*, *Symphytum*, and many other genera), and Fabaceae (genus *Crotalaria*) families [2,7,8]. PAs are composed of necine base esterified with necic acids normally at position(s) C-7 and/or C-9 (Fig. 1). The necine base is either saturated or possessed a double bond at the 1,2-position, rendering it potentially hepatotoxic and/or carcinogenic [2,3,9,10].

PAs have been detected in some food products of animal origin, including eggs, meat and milk [[11], [12], [13]], as well as of plant origin such as herbal products [[14], [15], [16], [17], [18], [19]] and honey [[19], [20], [21], [22], [23]]. In humans, exposure to PAs can occur through various routes, including accidental ingestion of contaminated food [24] and consumption of animal products derived from animals fed with PA-contaminated feed, or the consumption of herbal products prepared with PA plants. Consequently, PA intoxications have been reported in some developing countries, namely India, Jamaica, Egypt, Ethiopia, Iraq and South Africa [[25], [26], [27]]. The toxicity of PAs is primarily attributed to their ability to form reactive metabolites that can bind to cellular components and disrupt normal physiological processes [28]. The health implications of PA exposure range from acute toxicity to chronic conditions [10,18,29,30], including liver damage, cancer [31], and developmental abnormalities [32]. Prolonged exposure to PAs has been associated with hepatotoxicity [29,33,34].

Africa is home to a rich diversity of plant species, with significant number naturally producing PAs. It is therefore not surprising that the earliest reports of human PA poisoning on the continent were in 1920 in South Africa when many people in the Western Cape suffered from liver cirrhosis after eating bread made with wheat contaminated with *Senecio burchellii* DC [27,35]. Since then, similar PA poisonings have been reported in Egypt [26,[36], [37], [38], [39]]. Recently, in Africa, high levels of PAs have been detected in plant-derived products such as honey [18,20,40] and herbal remedies [14,18,27], while trace amounts have been found in maize grains cultivated on farmland previously dominated with PA plant, *Chromolaena odorata* [3].

PA contamination of foodstuffs poses additional challenges for African countries already grappling with other food safety issues, such as mycotoxins, pesticide residues, and microbial contaminants. Notwithstanding, there are very few studies on PA contamination of food products on the continent, thus exacerbating the urgency of addressing this issue. This review therefore aims to highlight the pressing issue of PA contamination of foodstuffs in Africa by providing a comprehensive and up-to-date overview of the current state of knowledge on the pathways of contamination, and implications for human health. Furthermore, the review provides a platform for discussing potential strategies and interventions to mitigate PA contamination to ensure the safety of food consumed on the continent.

## 2 Methodology

A comprehensive review was conducted from 2001 to 2023, focusing mainly on PA contamination of foodstuffs (e.g. grains, honey, teas and livestock products) in Africa. Peer-reviewed online databases of PubMed, Web of Science, Scopus, Science Direct and Google Scholar were searched using keywords such as: “pyrrolizidine alkaloid plants”, “pyrrolizidine alkaloid plants AND Africa” “pyrrolizidine alkaloid AND foodstuff contamination”, “pyrrolizidine alkaloid AND “mitigation strategies” and “pyrrolizidine alkaloid AND health implication”. A total of 211 studies were identified through database searches, of which 67 studies were excluded due to duplication and irrelevance to the study. As a consequence, 144 studies met the inclusion criteria and objectives of the review, out of which 96 studies were conducted on PA contamination of food and herbal products from other continents while only 48 studies emanated from Africa.

## 3 Current trends in PA contamination of foodstuffs in Africa

PA contamination of food can occur through various pathways at different stages of production and processing. The primary pathways through which PAs can contaminate food in Africa are as follows.

### 3.1 Uptake of pyrrolizidine alkaloids by food crops from contaminated soil

Common farming practices such as slash-and-burnt and slash-and-mulch have been found to contaminate the soil with PAs [3]. Food crops absorb PAs from the soil or water, resulting in their accumulation in various plant parts, including leaves, stems, grains and roots, thereby introducing the toxin into the food chain [[41], [42], [43], [44]]. In Ghana, trace amounts of PAs were recently detected in maize grains and leave (forage) as a result of these common agricultural practices [3], while in South Africa, contamination of commercial rooibos tea (*Aspalathus linearis* (Burm.f.) R.Dahlgren) has been linked to the lateral transfer of PAs from *Senecio angustifoliu*s, a PA plant, to a non- PA plant, rooibos tea [44,45]. This clearly demonstrates that food crops could take-up PAs from the soil in the immediate vicinity of a PA plant material as a result of common farming practices.

### 3.2 Co-harvesting herbs/forage with PA plant material

PA-containing weeds can contaminate human and animal food during cultivation, especially when herbs or forage which resemble the PA weeds are grown or harvested in their immediate vicinity [[46], [47], [48]]. This resemblance makes PA weeds easily overlooked by the farmers during harvesting, thus co-harvested with the herbs or forage [49]. Further unintended contamination can occur during harvesting when the seeds of PA weeds are co-harvested with edible grains [[50], [51], [52]]. This has previously resulted in a high case-fatality rate in Egypt [36] and Ethiopia [26,[37], [38], [39]] where locally produced grains were contaminated with the seeds of PA plants. Also, livestock feed and forage can become contaminated with PAs if they contain plant materials that have been infested with PA plants. Livestock consuming contaminated feed or forage can accumulate PAs in their tissues, which can subsequently enter the food chain through meat, milk, or eggs. Although there are no reports of animal-derived products contaminated with PAs in Africa, trace amounts have however been detected in these products in other continents [[11], [12], [13]].

### 3.3 Intentional use of PA plants in food preparations

**Fig. 2:**
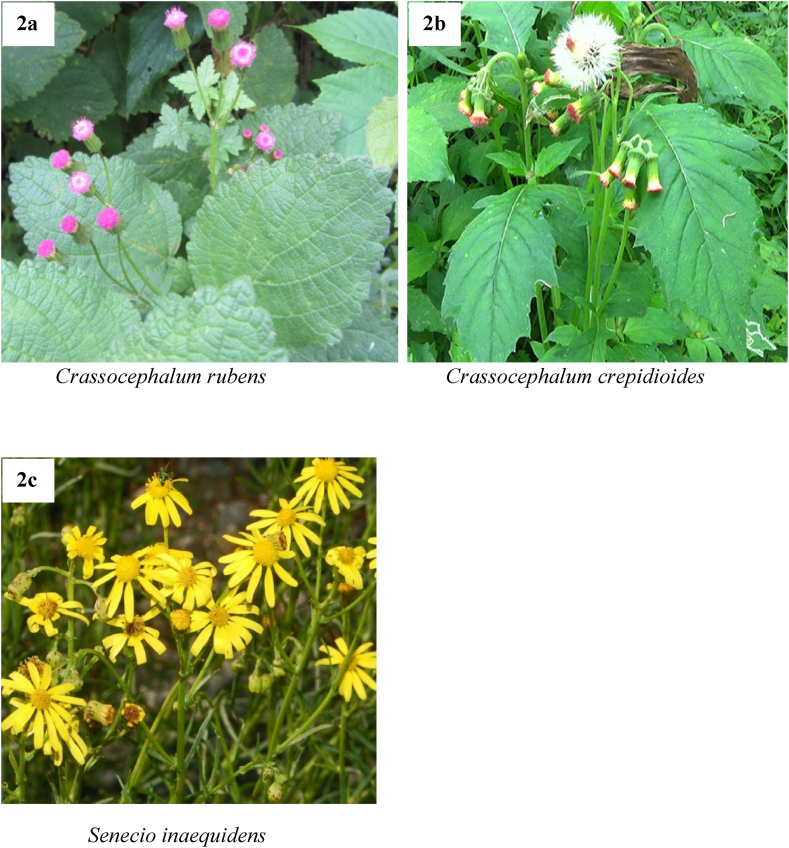
Views of pyrrolizidine alkaloid containing plants of the Asteraceae family commonly used in food preparations in Africa. Photos: **a**: Elke Faust; **b**: Matthew Thompson; **c**: Ettore Balocchi. Photos are licensed under Creative Commons Attribution-Noncommercial 4.0 International (CC BY-NC 4.0).

In Africa, herbs play a crucial role in flavouring and garnishing food as they provide significant macro- and micro-nutrients coupled with their perceived therapeutic and cultural significance [14,53,54]. However, some of these herbs have been found to contain hepatotoxic PAs. For instance, PA-containing plants such as *Crassocephalum crepidioides* (Benth.) S. Moore (Fig. 2a), *Crassocephalum rubens* (Fig. 2b) and *Senecio inaequidens* DC. (South African ragwort) (Fig. 2c) are commonly used fresh or dried for the preparation of sauces, soups and stews due to their purported health benefits [[55], [56], [57], [58]]. Particularly in Benin, *C. rubens* and *C. crepidioides* (popularly called “Gbolo”) are used as traditional leafy vegetables in sauces as they are believed to treat malaria, fever and indigestion [59,60]. Similarly, in Nigeria, the leaves and stems of *C. rubens* and *C. crepidioides* (popularly called “Ebolo”) are commonly consumed as vegetable in soups or sauces and for medicinal purposes [61]. In Uganda, however, the leaves are dried, chopped and cooked with peas or beans whereas in Malawi, they are cooked with groundnuts and tomatoes in soups [59]. In specific populations of southern Africa, the leaves of *S. inaequidens* are reportedly used as food due to its perceived anti-diabetic properties [58]. Again, intended adulteration of some herbal products with PA weeds by unscrupulous producers for economic benefits has recently been reported in Ghana [14]. Ingestion of PA-containing plants through this route has been implicated in the high rates of liver cancer and cirrhosis on the continent [39].

### 3.4 Foraging activities of honey bees

Bees inadvertently collect nectar and/or pollen from the flowers of PA-containing plants to produce honey. During the process, the PAs are transferred from the nectar or pollen into the honey, resulting in potential contamination of honey and other bee products. The honey is subsequently harvested and used as sweetener in breakfast meals, especially for children [20]. In Africa, high levels of PAs have been detected in honey and other bee products as a result of bees frequently foraging PA plants such as *Chromolaena odorata*, *Ageratum* spp., *Eupatorium* spp. *Crotalaria* spp., *Eupatorium* spp, *Echium* spp. and *Senecio* spp [20,62]. Furthermore, some herbal products in Ghana have been found to contain significant amounts of PAs owing to the use of honey as ingredient in the herbal preparations [14]. In view of the high levels of PAs detected in some honey on the continent, regular monitoring for PAs, especially for those harvested in the immediate vicinity of PA plants, is highly recommended [20].

## 4 Implications of food contaminated with pyrrolizidine alkaloid

The presence of PAs, specifically the 1,2-unsaturated PAs, in food products can have significant implications for human health and food safety. Since the liver is the main target organ for PA poisoning, consumption of food contaminated with PAs has been linked to liver damage, including the development of liver tumors and cancer [9,10,63]. In the liver, PAs are metabolized by cytochrome-P450 monooxygenase enzymes into reactive intermediates that can covalently bind to cellular macromolecules, such as proteins and DNA, to exert their toxicities [28,29,33,34]. The effects of this toxicity on human health depend on the level and duration of exposure. In acute toxicity, high amounts of PA-contaminated foods are consumed over a short period of time. This exposure has been associated with the development of veno-occlusive disease/sinusoidal obstructive syndrome (HVOD/SOS) [12], a condition characterized by the obstruction of small liver veins, which then progresses to liver fibrosis, cirrhosis and ultimately to cancer of the liver [39,64]. Chronic toxicity, on the other hand, involves the intake of smaller amounts of PAs over an extended period of time which has been linked to blocked of hepatic veins and breakdown of the surrounding liver tissues, eventually leading to death [29]. This exposure is usually associated with humans, especially children, who habitually consume herbal teas and honey containing small doses of PAs over a long period of time [14]. PAs can also have toxic effects on the lungs, which is the second target organ of PA toxicity, leading to pulmonary toxicities, including the formation of toxic metabolites, inflammation, tissue damage, and pulmonary hypertension.

## 5 Implementation of mitigation strategies for food safety

The mitigation of PA contamination of food is essential to ensure food safety and protect public health. Several strategies can be employed to minimize the presence of these toxic compounds in food sources in Africa.

### 5.1 Adoption of Good Agricultural Practices (GAPs)

Implementing and adhering to GAPs are crucial to prevent PA contamination at the cultivation stage, thereby guaranteeing safe and quality products. This includes planting food crops in PA-free soil where PA plants are completely excluded from the soil, crop rotation, and ensuring timely removal of weeds or other unwanted plants to minimize the growth of PA plants alongside food crops [65]. Additionally, hand harvesting techniques could be employed to minimize the incorporation of PA-containing plants or their parts into the harvested crop while avoiding the wild-harvesting of food crops [5]. Also, a search should be carried out on the fields before crop cultivation and prior to harvesting to identify PA weeds for proper and timely removal [66,67]. Again, to effectively minimize PA contamination of honey, all PA plants in the immediate vicinity of an apiary should completely be removed or the apiary relocated to fields free of PA plants to prevent bees foraging on their flowers for honey production [20].

### 5.2 Regulatory and quality control measures

Governments and regulatory bodies play a critical role in setting maximum allowable limits for PAs in food, conducting inspections, and enforcing compliance. For instance, the maximum allowable levels of PAs in certain foodstuffs, including dried herbs (fresh, frozen borage leaves, 750 μg/kg), pollen-based food supplements (500 μg/kg), food supplement (plant-derived, 400 μg/kg) and tea (dried *Camellia sinensis*, 150 μg/kg) have been determined by EU Commission Regulation (EU) 2020/2040 [68] to safeguard the health of consumers. Again, regular monitoring and surveillance programs can ensure that food products meet safety standards and mitigate contamination risks. Regular testing of food products for PA contamination, using reliable and validated analytical methods for PA detection, is essential to identify contaminated batches and potential sources so as to prevent them from reaching the consumers. For the determination of PA content, a sensitive and reliable technique such as liquid chromatography with tandem mass spectrometry detection (LC-MS/MS) is currently employed, which allow for the detection and quantification of the toxin [13,14,16,23]. Table 1 summarizes the PA content of some selected food items from different geographical regions determined by the various LC-MS/MS techniques. Rigorous quality control measures, including sampling and analysis, can help ensure compliance with regulatory limits and facilitate early detection of contamination while ensuring that suppliers adhere to PA safety standards.

**Table 1:** Selected food products from other continents analyzed with LC-MS/MS technique to determine their pyrrolizidine alkaloid content.

Food product	Highest PA level determined	Type of analysis	Country of origin of sample recording the highest PA level	References
**Plant-derived**				
Honey	1087 μg/kg	HPLC-QTRAP-MS/MS Positive ion mode and MRM mode	Uruguay	[69]
Honey	13019 μg/kg	HPLC-TQ-MS/MS ESI positive ion mode Column: C18	Netherlands	[70]
Honey	494.5 μg/kg	HRGC-Q-MS SIM mode Column: ZB-5MS (Arylene polymer) capillary column	Germany	[71]
Honey	4078 μg/kg	HPLC-IT-MS/MS ESI positive ion mode Column: C18 at 30 °C	non-EU countries	[72]
Honey	172 μg/kg	UHPLC-Q-MS ESI positive ion mode and SIM Mode Column: C8 at 34 °C	EU and non-EU countries	[73]
Honey	55 μg/kg	HPLC-QTRAP-MS/MS positive ion mode and MRM mode Column: C18 at 25 °C	Switzerland	[74]
Herbal medicine	3668 μg/kg	HPLC-TQ-MS/MS ESI positive ion mode and MRM mode Column: PFP at 35 °C	China	[75]
Herbal teas	1729 μg/kg	HPLC-QTRAP-MS/MS MRM mode Column: C18 at 25 °C	srael	[76]
Herbal teas	5668 μg/kg	HPLC-TQ-MS/MS ESI positive ion mode and MRM mode Column: C18 at 20 °C	Germany	[77]
Herbal medicine	7883 μg/kg	UHPLC-TQ-MS/MS MRM mode Column: C18 at 50 °C	China	[78]
Herbal food supplements	319 μg/kg	UHPLC-Q-Orbitrap-MS/MS ESI positive ion mode and HRMS mode Column: HSS T3 at 40 °C	USA	[79]
Herbal food supplements	8400 ng/g	UHPLC-QToF-MS/MS ESI positive ion mode and Allion MS/MS mode Column: C18 at 40 °C	USA, Mississippi	[80]
Herbs	1777 μg/kg	HPLC-TQ-MS/MS ESI positive ion mode and MRM mode Column: C18	Germany	[81]
Herbs	41 μg/kg	HPLC-QTRAP-MS/MS positive ion mode and MRM mode Column: C18 at 40 °C	Poland	[82]
Herbs and spices	24600 μg/kg	HPLC-TQ-MS/MS ESI positive ion mode and MRM mode Column: C18 at 30 °C	Turkey	[83]
**Animal-derived**				
Egg	885 μg/kg	HPLC-IT-MS/MS ESI positive ion mode Column: polar-reversed phase	Colombia	[84]
Milk	9.71 μg/L	UHPLC-QHQ-MS/MS MRM mode Column: C18 at 50 °C	Netherlands	[85]
Meat and eggs	392 μg/kg	UHPLC-TQ-MS/MS ESI positive ion mode and MRM mode Column: C18 at 50 °C	Netherlands	[86]

### 5.3 Education and awareness

Educating farmers and consumers in the entire value chain about the potential health risks associated with PA contamination is vital. Information on the importance of selecting food products from reliable sources and how to identify and minimize exposure to PAs is crucial. Also, consumers and producers should be encouraged to diversify their food choices and sources, respectively, to reduce overall PA exposure [14]. Training programs and awareness campaigns can help raise awareness of GAPs to minimize PA contamination risks, thereby safeguarding public health [5,65].

## 6 Discussion

The present review discussed the exposure to PAs in Africa and summarizes the current trends of food contamination and its health implications. The presence of PAs in food represents a significant threat to public health, particularly in Africa, where common farming practices have often led to the contamination of food crops. This therefore demands immediate attention from researchers, policymakers, food producers, and consumers to develop and implement effective strategies to detect, prevent, and mitigate PA contamination. These are essential to safeguarding the health and well-being of African populations and ensuring the availability of safe and nutritious food for all.

The review found that PA plants mostly foraged by bees for honey production in Africa, include *C. odorata*, *Ageratum* spp., *Eupatorium* spp. *Crotalaria* spp., *Eupatorium* spp, *Echium* spp. and *Senecio* spp. even though these plants were not the preferred choice for the bees. Out of the four current trends of PA contamination of foodstuffs identified in this review, “uptake of PAs by food crops from contaminated soil” and “foraging activities of honey bees” have often led to relatively low amounts of PAs in food products while significant levels of PAs have been detected in “co-harvesting herbs/forage with PA plant material” and “intentional use of PA plants in food preparations” contamination pathways. Moreover, it was discovered that the leaves of PA-containing plants, namely *C. crepidioides*, *C. rubens* and *S. inaequidens* are actively being used in the preparation of stews, sauces and soups due to their purported therapeutic benefits. Furthermore, three main mitigation strategies could be implemented to reduce the harmful effects of PAs. These include “adoption of Good Agricultural Practices”, “regulatory and quality control measures” and “education and awareness”. This review also found that studies are yet to be conducted to assess the levels of PAs in livestock products (e.g. milk, meat and eggs) on the continent.

On account of the health risk posed by the ingestion of foods contaminated with PAs, different analytical methods have been developed to detect and quantify PAs/PANOs in complex food matrices. For the extraction and quantification of PAs, reversed phase C_18_ SPE cartridges clean-up procedure and HPLC coupled with MS-instruments, respectively, have become the method of choice in recent years as they have been found to be sensitive, reliable and offers the advantage of a simultaneous detection of PAs and PANOs [14,87]. These techniques have been used to estimate the dietary exposures to toxic PAs and ascertain whether the levels of PA conform to regulatory limits.

Recognizing the potential dangers of PAs, regulatory authorities worldwide have taken measures to mitigate their harmful effects. These measures include setting maximum tolerable limits for PAs in food products, conducting regular monitoring and testing, and enforcing strict quality control measures throughout the food production chain. By combining these mitigation strategies, coupled with the adoption of GAPs and awareness creation, it is possible to reduce the incidence of PA contamination in food and protect consumers from potential health hazards. Collaboration between stakeholders, including farmers, food producers, regulators, and consumers, is essential to achieving effective mitigation measures and ensuring food safety.

## 7 Conclusions and future perspectives

The findings revealed that PA contamination of foodstuffs is a prevalent issue in several African countries, with varying degrees of contamination observed across different food types and geographical regions. This is partly due to the tropical, dry or temperate climatic zones of Africa which promote the growth of diverse botanicals that are invasive and/or herbivore deterrent mainly due to their alkaloid contents. This review further highlights the urgent need to monitor PAs and outlines intervention strategies to effectively manage and mitigate PA contamination in African food systems. It was also revealed that honey and other honey products are often contaminated with PAs while three PA plants from the Asteraceae family are actively being used in the preparation of stews, soups, and sauces in Benin, Nigeria, Uganda, Malawi, and southern Africa. The exposure to PAs, especially for frequent and high consumers of PA contaminated food products, over a prolonged period could pose a serious health risk due to their potential liver toxicity and carcinogenicity. For this reason, regulatory bodies on the continent are highly encouraged to establish maximum tolerable limits, as well as monitor food products (especially honey) regularly to protect the health of consumers. Also, as the data on thermal stability of PAs are limited, further studies are therefore required to ascertain the effects of different traditional cooking processes on the levels of PAs so as to obtain a real exposure assessment of the population on the continent. Further studies are required to ascertain the levels of PAs in livestock products (e.g., milk, meat and eggs), as well as in below-ground food crops (e.g., yam, cassava etc.), especially when they are cultivated on farmlands which previously contain PA plants.

## Funding

This research did not receive any specific grant from funding agencies in the public, commercial, or not-for-profit sectors.

## Ethics statement

Review and/or approval by an ethics committee was not needed for this study because it is a review paper and does not require human participants.

## Data availability statement

Data will be made available on request.

## Additional information

No additional information is available for this paper.

## CRediT authorship contribution statement

**Emmanuel Letsyo:** Writing – review & editing, Writing – original draft, Resources, Methodology, Formal analysis, Data curation, Conceptualization. **Felix Kwashie Madilo:** Writing – review & editing, Writing – original draft, Project administration, Methodology, Data curation, Conceptualization. **Liticia Effah-Manu:** Writing – review & editing, Writing – original draft, Validation, Investigation, Data curation, Conceptualization.

## Declaration of competing interest

The authors declare that they have no known competing financial interests or personal relationships that could have appeared to influence the work reported in this paper.

## References

[1] World Health Organization (Who) (2020). https://www.who.int/news-room/fact-sheets/detail/food-safety.

[2] Knutsen H.K., Alexander J., Barregärd L., Bignami M., Brűschweiler B., Ceccatelli S., Cottrill B., Dinovi M., Edler L., Grasl-Kraupp B., Hogstrand C., Hoogenboom L.R., Nebbia C.S., Oswald I.P., Petersen A., Rose M., Roudot A.-C., Schwerdtle T., Vleminckx C., Vollmer G., Wallace H., Gomez-Ruiz J.A., Binaglia M., EFSA CONTAM Panel (EFSA Panel on Contaminants in the Food Chain) (2017). Statement on the risks for human health related to the presence of pyrrolizidine alkaloids in honey, tea, herbal infusions and food supplements. EFSA J..

[3] Letsyo E., Adams Z.S., Dzikunoo J., Asante-Donyinah D. (2021). Uptake and accumulation of pyrrolizidine alkaloids in the tissues of maize (*Zea mays* L.) plants from the soil of a 4-year-old *Chromolaena odorata* dominated fallow farmland. Chemosphere.

[4] Zan K., Wang Z., Wang Y., Yu J.-D., Jin H.-Y., Ma S.-C. (2023). Distribution, determination method, risk assessment, and strategy of exogenous pyrrolizidine alkaloids in tea. Pharmacological Research-Modern Chinese Medicine.

[5] Alimentarius Codex (2014). http://www.fao.org/fao-whocodexalimentarius/download/standards/13794/CXP_074e_2014.pdf.

[6] García-Juan A., León N., Armenta S., Pardo O. (2023). Development and validation of an analytical method for the simultaneous determination of 12 ergot, 2 tropane, and 28 pyrrolizidine alkaloids in cereal-based food by LC-MS/MS. Food Res. Int..

[7] Schramm S., Kohler N., Rozhon W. (2019). Pyrrolizidine alkaloids: biosynthesis, biological activities and occurrence in crop plants. Molecules.

[8] Schrenk D., Gao L., Lin G., Mahony C., Mulder P.P.J., Peijnenburg A., Pfuhler S., Rietjens I.M.C.M., Rutz L., Steinhoff B., These A. (2020). Pyrrolizidine alkaloids in food and phytomedicine: occurrence, exposure, toxicity, mechanisms, and risk assessment - a review. Food Chem. Toxicol..

[9] Fu P.P. (2017). Pyrrolizidine Alkaloids: metabolic activation pathways leading to liver tumor initiation. Chem. Res. Toxicol..

[10] He Y., Zhu L., Ma J., Lin G. (2021). Metabolism-mediated cytotoxicity and genotoxicity of pyrrolizidine alkaloids. Archive of Toxicology.

[11] Mulder P.P., de Witte S.L., Stoopen G.M., van der Meulen J., van Wikselaar P.G., Gruys E., Groot M.J., Hoogenboom R.L.A. (2016). Transfer of pyrrolizidine alkaloids from various herbs to eggs and meat in laying hens. Food Addit. Contam. Part A Chem Anal Control Expo Risk Assess.

[12] Dusemund B., Nowak N., Sommerfeld C., Lindtner O., Schafer B., Lampen A. (2018). Risk assessment of pyrrolizidine alkaloids in food of plant and animal origin. Food Chem. Toxicol..

[13] Mulder P.P.J., Klijnstra M.D., Goselink R.M.A., van Vuuren A.M., Cone J.W., Stoopen G., Hoogenboom R. (2020). Transfer of pyrrolizidine alkaloids from ragwort, common groundsel and viper's bugloss to milk from dairy cows. Food Addit. Contam. Part A Chem Anal Control Expo Risk Assess.

[14] Letsyo E., Jerz G., Winterhalter P., Beuerle T. (2017). Toxic pyrrolizidine alkaloids in herbal medicines commonly used in Ghana. J. Ethnopharmacol..

[15] Letsyo E., Jerz G., Winterhalter P., Lindigkeit R., Beuerle T. (2017). Incidence of pyrrolizidine alkaloids in herbal medicines from German retail markets: risk assessments and implications to consumers. Phytother Res..

[16] Wiesner J. (2022). Regulatory perspectives of pyrrolizidine alkaloid contamination in herbal medicinal products. Planta Med..

[17] Fernández-Pintor B., Casado N., Morante-Zarcero S., Sierra I. (2023). Evaluation of the thermal stability and transfer rate of pyrrolizidine alkaloids during the brewing of herbal infusions contaminated with Echium vulgare and Senecio vulgaris weeds. Food Control.

[18] Letsyo E., Dzikunoo J., Dzah C.S., Adams Z.S., Asante-Donyinah D. (2023). Hepatic phytotoxins in herbal medicines: a review of Africa's pyrrolizidine alkaloid-containing plants, their traditional uses, contamination pathways and associated health risks. South Afr. J. Bot..

[19] Rizzo S., Celano R., Piccinelli A.L., Russo M., Rastrelli L. (2023). Target screening method for the quantitative determination of 118 pyrrolizidine alkaloids in food supplements, herbal infusions, honey and teas by liquid chromatography coupled to quadrupole orbitrap mass spectrometry. Food Chem..

[20] Letsyo E., Jerz G., Winterhalter P., Dubecke A., von der Ohe W., von der Ohe K., Beuerle T. (2017). Pyrrolizidine alkaloids in floral honeys of tropical Ghana: a health risk assessment. Food Addit. Contam. Part B Surveill.

[21] Celano R., Piccinelli A.L., Campone L., Russo M., Rastrelli L. (2019). Determination of selected pyrrolizidine alkaloids in honey by dispersive liquid-liquid microextraction and ultrahigh-performance liquid chromatography-tandem mass spectrometry. J. Agric. Food Chem..

[22] He Y., Zhu L., Ma J., Wong L., Zhao Z., Ye Y., Fu P.P., Lin G. (2020). Comprehensive investigation and risk study on pyrrolizidine alkaloid contamination in Chinese retail honey. Environ. Pollut..

[23] Brugnerotto P., Seraglio S.K.T., Schulz M., Gonzaga L.V., Fett R., Costa A.C.O. (2021). Pyrrolizidine alkaloids and beehive products: a review. Food Chem..

[24] Tesfaye W., Mayer R., Mulatu E., Assefa A. (2021). A review on pyrrolizidine alkaloids (PAs) and their potential health effects. EC Pharmacology and Toxicology.

[25] Botha C.J., Penrith M.L. (2008). Poisonous plants of veterinary and human importance in southern Africa. J. Ethnopharmacol..

[26] Molyneux R.J., Gardner D.L., Colegate S.M., Edgar J.A. (2011). Pyrrolizidine alkaloid toxicity in livestock: a paradigm for human poisoning?. Food Addit. Contam. Part A Chem Anal Control Expo Risk Assess.

[27] Wiedenfeld H. (2011). Plants containing pyrrolizidine alkaloids: toxicity and problems. Food Addit. Contam. Part A Chem Anal Control Expo Risk Assess.

[28] Abdelfatah S., Nass J., Knorz C., Klauck S.M., Kupper J.H., Efferth T. (2021). Pyrrolizidine alkaloids cause cell cycle and DNA damage repair defects as analyzed by transcriptomics in cytochrome P450 3A4-overexpressing HepG2 clone 9 cells. Cell Biol. Toxicol..

[29] Chen T., Mei N., Fu P.P. (2010). Genotoxicity of pyrrolizidine alkaloids. J. Appl. Toxicol..

[30] Chen L., Mulder P.P.J., Peijnenburg A., Rietjens I. (2019). Risk assessment of intake of pyrrolizidine alkaloids from herbal teas and medicines following realistic exposure scenarios. Food Chemistry and Toxicology.

[31] Sultana S., Ahmed S., Haque M.M., Hasan M. (2018). Pyrrolizidine alkaloids: potential role in the etiology of cancers, pulmonary and hepatic fibrosis, and congenital anomalies. Toxin Rev..

[32] EFSA (2011). Scientific Opinion on pyrrolizidine alkaloids in food and feed. EFSA J..

[33] Luo S., Chu J., Huang H., Yao K. (2018). Direct intrahepatic portocaval shunt for sinusoidal obstruction syndrome associated with hepatotoxicity of pyrrolizidine alkaloids. Biomedical Research International.

[34] Stegelmeier B.L., Colegate S.M., Brown A.W. (2016). Dehydropyrrolizidine alkaloid toxicity, cytotoxicity, and carcinogenicity. Toxins.

[35] Flade J., Beschow H., Wensch-Dorendorf M., Plescher A., Watjen W. (2019). Occurrence of nine pyrrolizidine alkaloids in *Senecio vulgaris* L. Depending on developmental stage and season. Plants.

[36] Stewart M.J., Steenkamp V. (2001). Pyrrolizidine poisoning: a neglected area in human toxicology. Ther. Drug Monit..

[37] Nibret E., Sporer F., Asres K., Wink M. (2009). Antitrypanosomal and cytotoxic activities of pyrrolizidine alkaloid-producing plants of Ethiopia. Journal of Pharmaceutical Pharmacology.

[38] Chiu C., Colleen Martin C., Woldemichael D., Selasie G.W., Tareke I., Luce R., Libanos G.G., Hunt D., Bayleyegn T., Addissie A., Buttke D., Bitew A., Vagi S., Murphy M., Seboxa T., Jima D., Debella A. (2016). Surveillance of a chronic liver disease of unidentified cause in a rural setting of Ethiopia: a case study. Ethiop. Med. J..

[39] Weldearegay K.T., Gebrekidan M.G., Gezahegne A.A. (2019). Health impact of hepatic-venous-occlusive disease in a small town in Ethiopia-Case study from Tahtay koraro district in Tigray region, 2017. PLoS One.

[40] Letsyo E., Ameka G. (2019). Major plants foraged by bees for honey production in Ghana: mapping of bee floral sources for the development of the apicultural industry. Grana.

[41] Yahyazadeh M., Nowak M., Kima H., Selmar D. (2017). Horizontal natural product transfer: a potential source of alkaloidal contaminants in phytopharmaceuticals. Phytomedicine.

[42] Selmar D., Wittke C., Beck-von Wolffersdorff I., Klier B., Lewerenz L., Kleinwachter M., Nowak M. (2019). Transfer of pyrrolizidine alkaloids between living plants: a disregarded source of contaminations. Environ. Pollut..

[43] Izcara S., Casado N., Morante-Zarcero S., Pérez-Quintanilla D., Sierra I. (2022). Miniaturized and modified QuEChERS method with mesostructured silica as clean-up sorbent for pyrrolizidine alkaloids determination in aromatic herbs. Food Chem..

[44] Hardie A.G., Olifant K., Smith J.F.N., Hoffman J.E. (2023). Scientific evidence of soil transfer of pyrrolizidine alkaloids originating from weed species to rooibos tea. South Afr. J. Bot..

[45] Van Wyk B.E., Stander M.A., Long H.S. (2017). *Senecio angustifolius* as the major source of pyrrolizidine alkaloid contamination of rooibos tea (*Aspalathus linearis*). South Afr. J. Bot..

[46] Mulder P.P.J., Lopez P., Castellari M., Bodi D., Ronczka S., Preiss-Weigert A., These A. (2018). Occurrence of pyrrolizidine alkaloids in animal- and plant-derived food: results of a survey across Europe. Food Addit. Contam. Part A Chem Anal Control Expo Risk Assess.

[47] Reinhard H., Zoller O. (2021). Pyrrolizidine alkaloids in tea, herbal tea and iced tea beverages- survey and transfer rates. Food Addit. Contam. Part A Chem Anal Control Expo Risk Assess.

[48] Steinhoff B. (2022). Pyrrolizidine alkaloid contamination in medicinal plants: regulatory requirements and their impact on production and quality control of herbal medicinal products. Planta Med..

[49] Casado N., Morante-Zarcero S., Sierra I. (2022). The concerning food safety issue of pyrrolizidine alkaloids: an overview. Trends Food Sci. Technol..

[50] Botha C.J., Lewis A., du Plessis E.C., Clift S.J., Williams M.C. (2012). *Crotalariosis equorum* ("jaagsiekte") in horses in southern Mozambique, a rare form of pyrrolizidine alkaloid poisoning. J. Vet. Diagn. Invest..

[51] Rouamba A., Ouédraogo V., Karama I., Compaoré M., Kiendrebeogo M. (2018). Ethno-medicinal use of *Crotalaria retusa* L. (Fabaceae), a pyrrolizidine alkaloid toxic plant. Int. J. Biochem. Res. Rev..

[52] Solofomalala A.H.D., Rajemiarimoelisoa C.F., Judicael R.L., Randrianarivo H.R., Rakoto D.A.D., Jeannoda V.L., Boumendjel A. (2021). Pyrrolizidine-derived alkaloids: highly toxic components in the seeds of *Crotalaria cleomifolia* used in popular beverages in Madagascar. Molecules.

[53] Steenkamp V., Stewart M.J., van der Merwe S., Zuckerman M., Crowther N.J. (2001). The effect of *Senecio latifolius* a plant used as a South African traditional medicine, on a human hepatoma cell line. J. Ethnopharmacol..

[54] Asres K., Sporer F., Wink M. (2007). Identification and quantification of hepatotoxic pyrrolizidine alkaloids in the Ethiopian medicinal plant *Solanecio gigas* (Asteraceae). Pharmazie.

[55] Heger T., Böhmer H.J. (2006). http://www.nobanis.org.

[56] Dimande A.F.P., Botha C.J., Prozesky L., Bekker L., Rösemann G.M., Labuschagne L., Retief E. (2007). The toxicity of *Senecio inaequidens* DC. J. S. Afr. Vet. Assoc..

[57] Roeder E., Wiedenfeld H. (2011). Pyrrolizidine alkaloids in plants used in the traditional medicine of Madagascar and the Mascarene islands. Pharmazie.

[58] Global Invasive Species Database (Gisd) (2022). http://www.iucngisd.org/gisd/species.php?sc=1458.

[59] Adjatin A., Dansi A., Eze C.S., Assogba P., Dossou-Aminon I., Akpagana K., Akoègninou A., Sanni A. (2012). Ethnobotanical investigation and diversity of Gbolo (*Crassocephalum rubens* (Juss. ex Jacq.) S. Moore and *Crassocephalum crepidioides* (Benth.) S. Moore), a traditional leafy vegetable under domestication in Benin. Genet. Resour. Crop Evol..

[60] Akakpo A.D.M., Achigan-Dako E.G. (2019). Nutraceutical uses of traditional leafy vegetables and transmission of local knowledge from parents to children in southern Benin. Agronomy.

[61] Adedayo B.C., Oboh G., Oyeleye S.I., Ejakpovi I.I., Boligon A.A., Athayde M.L. (2015). Blanching alters the phenolic constituents and in vitro antioxidant and anticholinesterases properties of fireweed (*Crassocephalum crepidioides*). Journal of Taibah University Medical Sciences.

[62] Edgar J.A., Roeder E., Molyneux R.J. (2002). Honey from plants containing pyrrolizidine alkaloids: a potential threat to health. J. Agric. Food Chem..

[63] Moreira R., Pereira D.M., Valentao P., Andrade P.B. (2018). Pyrrolizidine alkaloids: chemistry, pharmacology, toxicology and food safety. Int. J. Mol. Sci..

[64] Neuman M.G., Cohen L.B., Steenkamp V. (2017). Pyrrolizidine alkaloids enhance alcohol-induced hepatocytotoxicity in vitro in normal human hepatocytes. European Review for Medical and Pharmacological Sciences Sci.

[65] Joint Fao/Who Food Standards Programme (2014). https://www.fao.org/fao-who-codexalimentarius/codex-texts/codes-of-practice/en/.

[66] Neuman M.G., Cohen L.B., Opris M., Nanau R., Jeong H. (2015). Hepatotoxicity of pyrrolizidine alkaloids. J. Pharm. Pharmaceut. Sci..

[67] Yan X., Kang H., Feng J., Yang Y., Tang K., Zhu R., Yang L. (2016). Identification of toxic pyrrolizidine alkaloids and their common hepatotoxicity mechanism. Int. J. Mol. Sci..

[68] EU Commission Regulation (2020). https://eurlex.europa.eu/legalcontent/EN/TXT/PDF/?uri=%20CELEX:32020R2040&from=EN.

[69] Dübecke A., Beckh G., Lüllmann C. (2011). Pyrrolizidine alkaloids in honey and bee pollen. Food Addit. Contam..

[70] Kempf M., Wittig M., Reinhard A., von der Ohe K., Blacqui′ ere T., Raezke K.P., Michel R., Schreier P., Beuerle T. (2011). Pyrrolizidine alkaloids in honey: comparison of analytical methods. Food Addit. Contam..

[71] Cramer L., Beuerle T. (2012). Detection and quantification of pyrrolizidine alkaloids in antibacterial medical honeys. Planta Med..

[72] Griffin C.T., Danaher M., Elliott C.T., Kennedy D.G., Furey A. (2013). Detection of pyrrolizidine alkaloids in commercial honey using liquid chromatography–ion trap mass spectrometry. Food Chem..

[73] Martinello M., Cristofoli C., Gallina A., Mutinelli F. (2014). Easy and rapid method for the quantitative determination of pyrrolizidine alkaloids in honey by ultra performance liquid chromatography-mass spectrometry: an evaluation in commercial honey. Food Control.

[74] Kast C., Dübecke A., Kilchenmann V., Bieri K., Böhlen M., Zoller O., Beckh G., Lüllmann C. (2014). Analysis of Swiss honeys for pyrrolizidine alkaloids. J. Apicult. Res..

[75] Griffin C.T., Gosetto F., Danaher M., Sabatini S., Furey A. (2014). Investigation of targeted pyrrolizidine alkaloids in traditional Chinese medicines and selected herbal teas sourced in Ireland using LC-ESI-MS/MS. Food Addit. Contam..

[76] Shimshoni J.A., Duebecke A., Mulder P.P., Cuneah O., Barel S. (2015). Pyrrolizidine and tropane alkaloids in teas and the herbal teas peppermint, rooibos and chamomile in the Israeli market. Food Addit. Contam..

[77] Schulz M., Meins J., Diemert S., Zagermann-Muncke P., Goebel R., Schrenk D., Schubert-Zsilavecz M., Abdel-Tawab M. (2015). Detection of pyrrolizidine alkaloids in German licensed herbal medicinal teas. Phytomedicine.

[78] Chen L., Mulder P.P.J., Peijnenburg A., Rietjens I. (2019). Risk assessment of intake of pyrrolizidine alkaloids from herbal teas and medicines following realistic exposure scenarios. Food Chemistry and Toxicology.

[79] Vaclavik L., Krynitsky A.J., Rader J.I. (2014). Targeted analysis of multiple pharmaceuticals, plant toxins and other secondary metabolites in herbal dietary supplements by ultra-high performance liquid chromatography–quadrupole-orbital ion trap mass spectrometry. Anal. Chim. Acta.

[80] Avula B., Sagi S., Wang Y.H., Zweigenbaum J., Wang M., Khan I.A. (2015). Characterization and screening of pyrrolizidine alkaloids and N-oxides from botanicals and dietary supplements using UHPLC-high resolution mass spectrometry. Food Chem..

[81] Selmar D., Wittke C., Beck-von Wolffersdorff I., Klier B., Lewerenz L., Kleinwachter M., Nowak M. (2019). Transfer of pyrrolizidine alkaloids between living plants: a disregarded source of contaminations. Environ. Pollut..

[82] Kaczynski ′ P., Łozowicka B. (2020). A novel approach for fast and simple determination pyrrolizidine alkaloids in herbs by ultrasound-assisted dispersive solid phase extraction method coupled to liquid chromatography–tandem mass spectrometry. J. Pharmaceut. Biomed. Anal..

[83] Kaltner F., Rychlik M., Gareis M., Gottschalk C. (2020). Occurrence and risk assessment of pyrrolizidine alkaloids in spices and culinary herbs from various geographical origins. Toxins.

[84] Diaz G.J., Almeida L.X., Gardner D.R. (2014). Effects of dietary *Crotalaria pallida* seeds on the health and performance of laying hens and evaluation of residues in eggs. Res. Vet. Sci..

[85] Hoogenboom L.A.P., Mulder P.P., Zeilmaker M.J., Van den Top H.J., Remmelink G.J., Brandon E.F., Klijnstra M., Meijer G.A.L., Schothorst R., Egmond H.P.V. (2011). Carry-over of pyrrolizidine alkaloids from feed to milk in dairy cows. Food Addit. Contam..

[86] Mulder P.P., de Witte S.L., Stoopen G.M., van der Meulen J., van Wikselaar P.G., Gruys E., Groot M.J., Hoogenboom R.L.A. (2016). Transfer of pyrrolizidine alkaloids from various herbs to eggs and meat in laying hens. Food Addit. Contam. Part A Chem Anal Control Expo Risk Assess.

[87] Ma C., Liu Y., Zhu L., Ji H., Song X., Guo H., Yi T. (2018). Determination and regulation of hepatotoxic pyrrolizidine alkaloids in food: a critical review of recent research. Food Chem. Toxicol..

